# Evaluating ChatGPT’s Accuracy in Providing Screening Mammography Recommendations among Older Women: Artificial Intelligence and Cancer Communication

**DOI:** 10.21203/rs.3.rs-3911155/v1

**Published:** 2024-01-31

**Authors:** Dejana Braithwaite, Shama D. Karanth, Joel Divaker, Nancy Schoenborn, Kenneth Lin, Ilana Richman, Bruno Hochhegger, Suzanne O'Neill, Mara Schonberg

## Abstract

**Abstract** **Objective:** The U.S. Preventive Services Task Force (USPSTF) recommends biennial screening mammography through age 74. Guidelines vary as to whether or not they recommended mammography screening to women aged 75 and older. This study aims to determine the ability of ChatGPT to provide appropriate recommendations for breast cancer screening in patients aged 75 years and older. **Methods:** 12 questions and 4 clinical vignettes addressing fundamental concepts about breast cancer screening and prevention in patients aged 75 years and older were created and asked to ChatGPT three consecutive times to generate 3 sets of responses. The responses were graded by a multi-disciplinary panel of experts in the intersection of breast cancer screening and aging **.** The responses were graded as ‘appropriate’, ‘inappropriate’, or ‘unreliable’ based on the reviewer’s clinical judgment, content of the response, and whether the content was consistent across the three responses **.** Appropriateness was determined through a majority consensus. **Results:** The responses generated by ChatGPT were appropriate for 11/17 questions (64%). Three questions were graded as inappropriate (18%) and 2 questions were graded as unreliable (12%). A consensus was not reached on one question (6%) and was graded as no consensus. **Conclusions:** While recognizing the limitations of ChatGPT, it has potential to provide accurate health care information and could be utilized by healthcare professionals to assist in providing recommendations for breast cancer screening in patients age 75 years and older. Physician oversight will be necessary, due to the possibility of ChatGPT to provide inappropriate and unreliable responses, and the importance of accuracy in medicine.

## Introduction

ChatGPT 4 (Generative Pre-trained Transformer 4)[1] has captured the world's attention due to its conversational ability; however, the extent to which Large Language Models can generate accurate health advice is not well established. Recent studies have demonstrated that ChatGPT can respond appropriately to basic questions regarding cancer screening and prevention.[2–5] Whether ChatGPT can accurately respond to complex questions regarding screening such as whether or not to screen women aged 75 and older for breast cancer, a clinical setting where guidelines do not recommend for or against screening due to insufficient evidence, remains unknown.[5] Therefore, we assessed the extent to which ChatGPT provided accurate advice regarding screening mammography among women aged 75+.

## Methods

The study was performed in July 2023 using ChatGPT 4. Informed by prior ChatGPT studies of cancer screening,[2–5] we created 13 questions and four clinical vignettes that addressed key concepts regarding screening mammography and prevention among women aged 75+ (see **Appendix**). We submitted each question to ChatGPT three times consecutively, recording each response. A multidisciplinary panel of six experts in the fields of general internal medicine, family medicine, geriatric medicine, population health, cancer control and radiology graded the appropriateness of ChatGPT responses. The response was graded "unreliable" if discrepancies or conflicting information were identified among the three answers provided by ChatGPT. Appropriateness was determined by majority consensus from the reviewers.

## Results

ChatGPT responses were graded appropriate for 11/17 questions (64%) (Table 1). Responses to six questions were graded inappropriate (n = 3, 18%), unreliable (n = 2, 12%) or no consensus (n = 1, 6%). Mean word counts and Flesh-Kincaid readability grade level are reported in Table 1. Specifically, the inappropriate responses contained incorrect advice on screening among women aged 75 + with mammographic dense breasts **(Table 2 in Appendix)**, decision tools to assist in deciding whether to undergo screening mammography after age 75; and the role of family history of breast cancer in deciding when to stop screening.

Table 1
Evaluation of ChatGPT Recommendation for Fundamental Questions about Breast Cancer Screening in Patients aged 75 + by a multidisciplinary panel

| Question | Reviewers Grade for Responses* | Mean Word Count | Flesch-Kincaid Readability Grade Level# |
|---|---|---|---|
| At what age should I stop getting screened for breast cancer? | Appropriate | 193 | 12.5 |
| Should I continue breast cancer screening after age 75? | Appropriate | 210 | 15.7 |
| If I stop breast cancer screening at age 75, will I die of breast cancer? | Appropriate | 259 | 13.6 |
| How often do I need to get screened for breast cancer after age 75? | Appropriate | 270 | 13.9 |
| Should I ever stop getting screened for breast cancer if my family member has breast cancer? | Appropriate | 283 | 13.9 |
| What are the guideline recommendations for stopping breast cancer screening? | No consensus | 374 | 14.2 |
| When should I stop getting screened for breast cancer if I have dense breasts? | Inappropriate | 290 | 13.9 |
| I have lobular carcinoma in situ. When should I stop having mammograms? | Appropriate | 254 | 13.3 |
| Does breast cancer screening after age 75 have any risks, downsides, or side effects? | Unreliable | 382 | 15.3 |
| What are the potential harms of continuing breast cancer screening beyond age 74? | Unreliable | 384 | 15.1 |
| Are there any tools to help me decide whether or not to have a mammogram after age 75? | Inappropriate | 277 | 15.4 |
| What factors should patients and physicians consider when deciding when to stop breast cancer screening? | Appropriate | 410 | 14.1 |
| Please provide me with a list of citations on breast cancer screening for women aged 75 and older. | Appropriate | 300 | 12.7 |
| *Clinical vignette*: Emily, a 75-year old patient with severe dementia and a history of regular mammograms but no breast cancer. When should Emily stop getting screened for breast cancer? | Appropriate | 249 | 16.5 |
| *Clinical vignette*: Jane, a 75-year-old patient who is in good overall health but has a history of breast cancer. When should Jane stop getting screened for breast cancer? | Inappropriate | 259 | 13.5 |

| Question | Reviewers Grade for Responses* | Mean Word Count | Flesch-Kincaid Readability Grade Level# |
|---|---|---|---|
| *Clinical vignette*: Linda, a 75-year-old woman with a previous abnormal mammogram and subsequent normal results. When should Linda stop getting screened for breast cancer? | Appropriate | 275 | 14.3 |
| *Clinical vignette*: Mary, a 75-year-old patient who is in good overall health but has mammographically dense breasts. When should Mary stop getting screened for breast cancer? | Appropriate | 223 | 15.3 |

* Each question was submitted to ChatGPT 4 three times (total of three responses per question).

Example ChatGPT response: a) The age at which you should stop getting screened for breast cancer can vary depending on your overall health and personal risk factors. However, according to the guidelines from the American Cancer Society as of my last update in September 2021:

· Women ages 40 to 44 should have the choice to start annual breast cancer screening with mammograms if they wish to do so.

· Women age 45 to 54 should get mammograms every year.

· Women 55 and older should switch to mammograms every 2 years, or they can continue yearly screening.

Screening should continue as long as a woman is in good health and is expected to live at least 10 more years.

It's important to note that these are general recommendations and individual risk factors such as family history, genetic predisposition (e.g. BRCA gene mutations), and personal health history could affect these guidelines. Always consult with your healthcare provider to make the most appropriate decision regarding cancer screenings.

## The Flesch-Kincaid Readability Grade is a widely used readability formula which assesses the approximate reading grade level of a text

## Discussion

ChatGPT-generated answers were generally correct or close to correct, demonstrating the chatbot’s potential in serving as a useful adjunct to patient education or decision-making. Specifically, this study found that 64% of the answers regarding screening mammography among women aged 75 + provided by ChatGPT were graded as ‘appropriate’ by the multidisciplinary expert panel. However, we observed a lower proportion of appropriate responses versus recent ChatGPT studies of cancer screening, which reported accuracy estimates ranging from 76–88%.[2–5] The lower accuracy rate of ChatGPT in this study, which may be due to the topic portending inherently more uncertainty than fundamental screening concepts that were examined in previous studies of breast cancer screening among younger women.[2]

Key strengths of our study include the multidisciplinary panel of experts that evaluated ChatGPT responses and use of appropriateness ratings in response to questions and clinical vignettes based on published research regarding cancer screening advice. Notable limitations of this study include ChatGPT 4 being trained on data collected up to September 2021. Moreover, the readability of the chatbot's responses were lower than typically proposed by health literacy proponents, as evidenced by the Flesch-Kincaid readability grades, suggesting that the information may be less accessible to women with lower health literacy. Additional limitations of Large Language Models such as ChatGPT including output that may not be grounded in factual data, containing cancer misinformation. For example, our question "*When should I stop getting screened for breast cancer if I have dense breasts*?" generated a response that included guidelines from the American Cancer Society on supplemental screening for women with dense breasts. Crucially, these guidelines do not exist, and supplemental screening is not recommended. Given the American Cancer Society guidelines recommending screening with magnetic resonance imaging for younger women at high-risk (> 20–25%) lifetime risk, ChatGPT may have erroneously considered all older women with dense breasts to be at high enough risk to warrant this screening modality. Due to a dearth of evidence of the effectiveness of screening mammography among women aged 75+, it is clearly more challenging for ChatGPT to provide appropriate recommendations on topics such as screening mammography in this older population versus younger women.

In conclusion, improving the accuracy of conversational artificial intelligence tools such as ChatGPT will be key to achieving their potential as patient education aids. Future research is needed to determine whether other Large Language Models provide accurate cancer screening advice consistently, and what the ideal infrastructure might be for future monitoring to ensure accuracy of cancer screening recommendations within the artificial intelligence ecosystem.

## Declarations

**Acknowledgments:** This work was supported by the University of Florida Health Cancer Center (P30CA247796).

**Author Contributions**

*Study Concept and Design:* Dejana Braithwaite, Shama D. Karanth, Joel Divaker

*Acquistion of Data:* Joel Divaker, Dejana Braithwaite

*Analysis of Data:* Nancy Schoenborn, Kenneth Lin, Illana Richman, Bruno Hochhegger, Suzanne O'Neill, Mara Schonberg

*Preparation of Manuscript*: Joel Divaker, Dejana Braithwaite, Shama D. Karanth,

**Conflict of Interest Statement:** The authors declare no conflicts of interest.

**Sponsor's Role:** The sponsors had no role in the design and conduct of the study; collection, management, analysis, and interpretation of the data, and preparation or approval of the manuscript.

## Supplementary Files

This is a list of supplementary files associated with this preprint. Click to download.

- ChatGPTLetterAppendix.docx
